# Evaluation of the chemical composition and nutritional value of lettuce (*Lactuca sativa* L.) biofortified in hydroponics with iodine in the form of iodoquinolines

**DOI:** 10.3389/fpls.2023.1288773

**Published:** 2023-11-23

**Authors:** Agnieszka Dyląg, Sylwester Smoleń, Anna Wisła-Świder, Iwona Kowalska, Olga Sularz, Joanna Krzemińska, Joanna Pitala, Aneta Koronowicz

**Affiliations:** ^1^ Department of Human Nutrition and Dietetics, Faculty of Food Technology, University of Agriculture in Krakow, Krakow, Poland; ^2^ Department of Plant Biology and Biotechnology, Faculty of Biotechnology and Horticulture, University of Agriculture in Krakow, Krakow, Poland; ^3^ Department of Chemistry, Faculty of Food Technology, University of Agriculture in Krakow, Krakow, Poland; ^4^ Laboratory of Mass Spectrometry, Faculty of Biotechnology and Horticulture, University of Agriculture in Krakow, Krakow, Poland

**Keywords:** 4-hydroxy-8-iodo-3-quinolinecarboxylic acid, 5,7-diiodo-8-quinolinol, 5-chloro-7-iodo-8-quinolinol, 8-hydroxy-7-iodo-5-quinolinesulfonic acid, enrichment efficiency, iodine deficiency, potassium iodate, sustainable horticulture

## Abstract

Iodine deficiency in the diet creates the need to search for innovative, more sustainable and more effective strategies for enriching food with this microelement. The adopted research hypothesis assumed that the use of organic forms of iodine for supplementation of lettuce (*Lactuca sativa* L.), compared to mineral iodine, has a more favorable effect not only on the concentration of iodine, but also on the yield and the content of other chemical components determining its nutritional and health-promoting value. Lettuce was planted in a nutrient film technique (NFT) hydroponic study in a greenhouse. The following application of iodine compounds (all in 5 µM molar mass equivalents) were tested in the studies: control (without of iodine application); potassium iodate (positive iodine control), 8-hydroxy-7-iodo-5-quinolinesulfonic acid, 5-chloro-7-iodo-8-quinolinol, 5,7-diiodo-8-quinolinol and 4-hydroxy-8-iodo-3-quinolinecarboxylic acid. In this work, it was shown for the first time that iodoquinolines can be 1) a source of iodine for plants; 2) they have a biostimulating effect on their yielding and 3) they increase the resistance of crops to stress (due to a significant increase in the level of polyphenolic compounds). Lettuce with the addition of 8-hydroxy-7-iodo-5-quinolinesulfonic acid was characterized by the highest content of iodine, which was 221.7 times higher than in control plants. The weight gain of the whole plant was particularly visible in the case of lettuce enriched with 5-chloro-7-iodo-8-quinolinol and amounted to 26.48% compared to the control. Lettuce biofortified with iodine in the form of iodoquinolines can successfully become part of a sustainable diet based on plant products, which has a low impact on the environment and contributes to the long-term good health of an individual or community. Reducing iodine deficiency through the use of organoiodine compounds can help achieve the sustainability goal of eliminating hidden hunger, improving nutritional status and promoting sustainable agriculture.

## 1 Introduction

Ensuring food security is a real problem, given climate change and the rapid increase in food demand for an ever-growing population estimated to be around 10.4 billion by 2100 (Adam, 2022). Intensified anthropopressure and extreme climatic phenomena have a negative impact on food production, which is manifested by a decrease in yields and their quality, as well as a growing loss of agricultural land (FAO et al., 2015). In addition, the factor influencing the reduction of the content of minerals and vitamins in crop plants was the intensification of agriculture, which took place in the second half of the last century during the so-called green revolution. The introduction of high-yielding varieties of crops into production inadvertently contributed to the depletion of minerals in food and, consequently, to nutritional deficiencies in the diet of the population (Khoshgoftarmanesh et al., 2010). Currently, one of the greatest challenges of the modern world is to reduce malnutrition in all its forms, often referred to as the triple burden of malnutrition (Meybeck and Gitz, 2017; Lowe, 2021). It includes: 1) insufficient supply of energy in the diet, i.e. chronic hunger, which affected 828 million people in 2021 (von Grebmer et al., 2022), 2) deficiencies of nutrients, such as primarily iodine, iron, zinc and vitamin A (hidden hunger), from which people suffer around 3 billion of the world’s population (Kiran et al., 2022); and 3) over-nutrition leading to overweight and obesity affecting more than 2 billion adults (Searchinger et al., 2019).

On September 25, 2015, the United Nations General Assembly adopted a resolution - Transforming our world: the 2030 Agenda for Sustainable Development, whose goals and objectives are based on an extremely ambitious vision of the world focused on change. According to this vision, all people have, above all, access to sufficient, safe, nutritious and affordable food and sustainable production and consumption patterns. Among the 17 Sustainable Development Goals, Goal 2 aims to end hunger and all forms of malnutrition by 2030, and ensure food and nutrition security by promoting sustainable agriculture (United Nations, 2015). Malik and Maqbool (2020) emphasized the need for biofortification of crops as a method necessary to achieve the above goal. Biofortification is a cost-effective and sustainable agricultural strategy that aims to increase the concentration or bioavailability of essential elements in the edible parts of plants, minimizing the risk associated with the presence of toxic metals and thus reducing malnutrition (Carvalho and Vasconcelos, 2013; Bouis and Saltzman, 2017). Agronomic biofortification, biofertilization and optimized use of fertilizers are now widely used methods and techniques worldwide (Koç and Karayiğit, 2022). Biofortification of staple crops can help alleviate micronutrient malnutrition or “hidden hunger” among resource-poor populations, especially in developing countries (Wakeel and Labuschagne, 2021).

Insufficient iodine intake, which affects around 2 billion people worldwide (Ma et al., 2022), leads to a series of dysfunctions called iodine deficiency disorders (IDD). They result from insufficient secretion of thyroid hormones (3,5,3’-triiodo-L-thyronine; 3,5,3’,5’-tetraiodo-L-thyronine), the characteristic symptom of which is enlargement of the thyroid gland (goiter) (Hatch-McChesney and Lieberman, 2022). However, the most serious consequences include neurodevelopmental disorders, memory and cognitive impairments, mental disorders and infant mortality, which affects the quality of life as well as economic productivity (Khoshgoftarmanesh et al., 2010).

Disruptions in food supply chains and the economic downturn caused by the COVID-19 pandemic have affected food systems around the world and threatened people’s access to food, making Sustainable Development Goal 2 even more remote (Abidoye et al., 2021). As such, urgent, short-term action is needed to prevent hunger from rising, and a transformation of food systems is needed to ensure a healthy and sustainable future for all (WHO, 2021). This means not only reducing greenhouse gas emissions in agriculture and throughout the food chain, but also changing the diet of the population to an environmentally sustainable diet (Macdiarmid, 2022). Changing the current dietary patterns based on animal products to a properly balanced diet in which high-quality plant products play a significant role may contribute not only to limiting global warming, stopping environmental degradation and the spread of diet-related diseases, but also to improving global food security (Keding et al., 2013; Lombardi et al., 2021). Moreover, this change is dictated by concern for the fate of the planet and future generations of its inhabitants (Macdiarmid, 2022).

Most of the previous works on iodine biofortification focused mainly on the use and impact of inorganic forms of iodine (potassium iodide and potassium iodate) on plants. It is worth noting that both mineral and organic iodine compounds are present in the soil, where this element is covalently bound to the aromatic ring (Halka et al., 2019). Iodoquinolines used for the innovative biofortification of lettuce are quinoline derivatives that show a wide spectrum of biological activity, including anti-inflammatory, antioxidant, anticancer, antiviral, antibacterial, antifungal and antiprotozoal effects. Quinoline or benzo[*b*]pyridine is a heterocyclic aromatic compound containing a nitrogen atom in its structure (Matada et al., 2021).

Lettuce (*Lactuca sativa* L.) is one of the most suitable plants for biofortification studies, because iodine uptake and absorption capacity is much higher in leafy vegetables than in fruit vegetables. In addition, it is usually eaten raw, eliminating the risk of iodine loss during cooking, and can be grown year-round (Gonzali et al., 2017). Due to its extensive geographical distribution, lettuce is one of the most popular and consumed leafy vegetables in the world, with China being the largest producer, accounting for more than half of the world’s production (Kim et al., 2016).

The hypothesis of these studies assumes that the use of organic iodine compounds (iodoquinolines) for biofortification of lettuce, compared to mineral iodine (potassium iodate), has a more favorable effect not only on the concentration of iodine, but also on the content of other chemical components (including secondary metabolites), which determine their nutritional and health-promoting value. In addition, iodoquinolines presumably can have a biostimulating effect on the growth of lettuce plants.

The aim of this study was to examine the effectiveness of the use of iodoquinolines [8-hydroxy-7-iodo-5-quinolinesulfonic acid, 5-chloro-7-iodo-8-quinolinol, 5,7-diiodo-8-quinolinol, 4-hydroxy-8-iodo-3-quinolinecarboxylic acid] and potassium iodate (KIO_3_) for the biofortification of lettuce with iodineby assessing its chemical composition (including the content of selected bioactive compounds) and determining the effect of these compounds on the growth of lettuce plants.

## 2 Materials and methods

### 2.1 Synthesis and physico-chemical characteristics of 5,7-diiodo-8-quinolinol

Two of iodoquinolines were *de novo* synthesized for the study: 5,7-diiodo-8-quinolinol and 4-hydroxy-8-iodo-3-quinolinecarboxylic acid.

The classic synthesis of 5,7-diiodoquinolinol described by Das and Mukherji (1957) was modified by using microwave conditions that optimize the reaction solvent, time and temperature. Molar amounts of substrates 8-hydroxyquinoline: free iodine: perhydrol - 1: 1.1: 0.2 in ethanol solution were heated in a microwave field for 5 minutes, which allowed to shorten the reaction time by 35 minutes (**Figure S1**). The solid product was filtered off, washed with hot water and dissolved in 0.1 M NaOH. The solution was filtered and the clear filtrate precipitated with a slight excess of 0.1 M HCl. The crude product was filtered off, washed with hot water and dried, then recrystallized from xylene for purification. The product was obtained in the form of beige needles and its structure was confirmed by ^1^H NMR, ^13^C NMR analysis.

^1^H NMR and ^13^C NMR spectra were recorded on a FT-NMR JEOL spectrometer at 500 and 125 MHz, respectively, with TMS as an internal standard (**Figures S2A, B**).

The synthesis and physicochemical data of the 4-hydroxy-8-iodo-3-quinolinecarboxylic acid will be published after the patent application is filed.

### 2.2 Plant material and treatments applied before harvest

In a spring season *Lactuca sativa* L. var. *capitata* cv. ‘Melodion’ was planted in a nutrient film technique (NFT) hydroponic study in a greenhouse. The experiment was performed on the campus of the University of Agriculture in Kraków, Poland (50°05’04.1”N 19°57’02.1”E). The seeds were sown in the middle of February. Seeds were sown into 96-cell propagation trays with 32x32x40 mm sized cells filled with peat substrate mixed with sand (1:1 v/v). Seedlings of 4-5 true leaves (in the middle of March) were transplanted into the NFT system. Seedlings were placed into holes (spaced 25 cm apart) of styrofoam slabs filling NFT beds - a “dry hydroponic” method of cultivation without substrate.

After transplanting, plants were watered for 1 minute at 5-minute intervals during the day between 5.00 and 19.00 and during the night between 1.00 and 2.00. The nutrient solution used for the cultivation contained the following amounts of macro- and microelements (mg dm^-3^): N-120, P-40, K-170, Mg-35, Ca-15, Fe-1.5, Mn-0.55, Zn-0.2, B-0.2, Cu-0.09 and Mo-0.04. At the beginning of lettuce cultivation in NFT system the EC of nutrient solution of all treatments was 1.75 mS cm^-1^ and the pH of all nutrient solutions was adjusted to 5.70 with the use of 38% nitric acid – according to our previous studies with lettuce in the same hydroponic system (Smoleń et al., 2019).

Studies included the introduction into the nutrient solution iodine organic compounds as iodoquinolines form and inorganic form of iodine as KIO_3_ − reference treatment as positive control to iodoquinolines. The following treatments were tested in the studies: 1) Control (without of iodine application), 2) KIO_3_, 3) 8-hydroxy-7-iodo-5-quinolinesulfonic acid, 4) 5-chloro-7-iodo-8-quinolinol, 5) 5,7-diiodo-8-quinolinol and 6) 4-hydroxy-8-iodo-3-quinolinecarboxylic acid. Application of all compounds started at the rosette stage (5–6 true leaves) in concentrations 5 µM (molar mass equivalents of each compounds). The nutrient solution (containing the compounds tested) was applied continually during lettuce cultivation until harvest of lettuce plants. After introducing the tested substances into the nutrient solutions, the level of nutrient medium in the tanks was not replenished or replaced during whole cultivation period. The experiment consisted of 4 replicates in a randomized block design. There were 10 plants per replicate and 40 plants per combination (240 plants per experiment).

For each treatment, 650 dm^3^ of nutrient solution were stored in separate containers and periodically administered to the cultivation slabs. The frequency of watering was adjusted for the growth stage of lettuce and weather conditions. Plants were cultivated in the recirculating system of nutrient solution without a disinfection system. Plants received the same nutrient solutions throughout the entire period. Lettuce harvest, followed by the assessment of head and root weight and collection of leaf samples, was conducted at the beginning of May. According to the procedure of Smoleń et al. (2021) when lettuce heads were harvested by pipette a secretion produced as a result of root pressure [white secretion (RootSec) on the surface of the root neck] was collected. The secretion was collected for the determination of the chemical forms of iodine compounds transported from roots to the above-ground parts of plants.

### 2.3 Analyzes in lyophilized material

Fresh lettuce leaves were frozen and then freeze-dried using a Christ Alpha 1-4 freeze dryer (Martin Christ Gefriertrocknungsanlagen GmbH, Germany). Lyophilized samples were ground in a laboratory mill (FRITSCH Pulverisette 14; FRITSCH GmbH, Idar-Oberstein, Germany) and stored in sealed polyethylene bags (at -20°C) for further analysis.

In the freeze-dried material, the basic chemical composition was assessed, the concentration of total iodine and total nitrogen as well as micro- and macroelements were determined. The content of B vitamins, total polyphenol content and antioxidant activity were also determined.

#### 2.3.1 Basic chemical composition

Standard methods of the Association of Official Analytical Chemists (Horwitz and Latimer, 2006) were used to determine the basic chemical composition, i.e. total protein, crude fat, total dietary fiber and ash. Total protein content was determined by the Kjeldahl method (AOAC method No. 950.36) using a Kjeltec 2200 analyzer (Foss Tecator AB, Höganäs, Sweden). Crude fat content was assessed by multiple continuous extraction - Soxhlet (AOAC method No. 935.38) using a Soxtec Avanti 2050 Auto System (Foss Tecator AB, Sweden). Total dietary fiber content was determined using the Total Dietary Fiber Assay Kit (Megazyme, Sydney, Australia), according to AOAC method No. 991.43. The ash content was determined by burning the samples in a muffle furnace (AOAC method No. 930.05). The following formula was used to calculate the content of available carbohydrates:


available carbohydrate content=100−(total protein content+crude fat content+ash content+total dietary fiber content)

#### 2.3.2 Determination of total iodine content

Total iodine content in freeze-dried samples of lettuce leaves was assessed by inductively coupled plasma mass spectrometry (ICP-MS/MS) using a triple quadrupole spectrometer (iCAP TQ ICP-MS; ThermoFisher Scientific, Bremen, Germany), after alkaline extraction of the samples tetramethylammonium hydroxide (TMAH). The analysis was performed according to the procedure described by Smoleń et al. (2019).

#### 2.3.3 Determination of the content of macro- and microelements

Total nitrogen content was determined by the Kjeldahl method using a Foss Digestor 2020 mineralization furnace by TecatorTM and a Velp UDK 139 semi-automatic distillation unit. Concentration of P, S, Na, K, Mg, Ca, Fe, Cu, Zn, Mn, Mo and B were determined by inductively coupled plasma highly dispersive optical emission spectrometry (ICP-OES) according to the method described by Kalisz et al. (2019). Before measuring the elements on ICP-OES, the samples were mineralized using the following method: 0.5 g of dried samples were placed in 55 ml TMF vessels and mineralized in 10 ml of 65% super pure HNO_3_ (Merck, no. 100443.2500) in a Mars 5 Xpress microwave digestion system (CEM, USA). The mineralization procedure was as follows: the time required to reach 200°C (15 minutes), followed by the time to maintain the temperature at 200°C (20 minutes). After cooling, the samples were quantitatively transferred to 25 ml volumetric flasks with redistilled water. The content of the above-mentioned elements was determined using an ICP-OES spectrometer (Prodigy Teledyne Leeman Labs, USA).

#### 2.3.4 Determination of the content of B-group vitamins

The content of B vitamins, such as thiamine hydrochloride (vitamin B1), riboflavin (vitamin B2), nicotinic acid (vitamin B3), nicotinamide (vitamin PP), pantothenic acid (vitamin B5), pyridoxine hydrochloride (vitamin B6) and folic acid (vitamin B9) was determined by high performance liquid chromatography coupled with tandem mass spectrometry and electrospray ionization (HPLC-ESI-MS/MS), according to the procedure previously described by Smoleń et al. (2022).

#### 2.3.5 Determination of total polyphenol content and antioxidant activity

Methanolic extracts for the determination of total polyphenol content and antioxidant activity were prepared according to the procedure previously described by Pellegrini et al. (2003).

The content of total polyphenols was determined using the Folin-Ciocalteu reagent as reported by Swain and Hillis (1959).

Antioxidant activity was determined using the cation radical ABTS^•+^ (2,2’-azino-bis(3-ethylbenzothiazoline-6-sulfonic acid)) according to the method described by Re et al. (1999) and the results obtained are expressed as µmol Trolox per g of dry weight.

Both analyses were performed using a UV-1800 spectrophotometer (RayLeigh, Beijing Beifen-Ruili Analytical Instrument Co., Ltd., Beijing, China).

### 2.4 Analyzes in fresh material

Immediately after harvesting, whole plants and lettuce heads were weighed, and then the dry matter content in the leaves was determined using the drying method at 105°C. In the fresh material, the concentration of quinoline, all tested iodoquinolines, iodide ions, L-ascorbic acid, dehydroascorbic acid, nitrogen compounds and chlorides was also determined.

#### 2.4.1 Determination of the content of quinoline, iodoquinolines and iodide ions in plant samples and RootSec

The method Smoleń et al. (Smoleń et al. 2021; Smoleń et al., 2023) for extraction and methods of measurement for determination of iodine-organic compounds (8-hydroxy-7-iodo-5-quinolinesulfonic acid, 5-chloro-7-iodo-8-quinolinol, 5,7-diiodo-8-quinolinol and 4-hydroxy-8-iodo-3-quinolinecarboxylic acid) in roots, leaves and RootSec, by LC-MS/MS technique, was used. Subject to the following: only for the analysis of 8-hydroxy-7-iodo-5-quinolinesulfonic acid we used a separate modification of the chromatographic method described by Krzemińska et al. (2023).

For determination of I^-^ ions in roots, leaves and RootSec the HPLC-ICP-MS/MS technique, was used. An analytical procedure Smoleń et al. (Smoleń et al. 2021; Smoleń et al., 2023) was used.

#### 2.4.2 Determination of the content of L-ascorbic and dehydroascorbic acid

The content of L-ascorbic acid (AA) and dehydroascorbic acid (DHA) in fresh leaves was analyzed by capillary electrophoresis using a diode array detector (DAD), according to the metod described by Smoleń et al. (2022). The analyses were performed using the PA 800 Plus capillary electrophoresis system (Beckman Coulter, Indianapolis, IN, USA).

The DHA content was determined after adding 50 mM dithiothreitol (DTT) to the supernatants after the second centrifugation, according to the method of Dresler and Maksymiec (2013). The DHA level was calculated by subtracting the initial AA concentration from the total AA concentration obtained after DHA reduction.

#### 2.4.3 Determination of the content of nitrogen compounds and chlorides

The concentration of ammonium ion (NH_4_^+^), nitrate (III) (NO_2_^-^), nitrate (V) (NO_3_^-^) and chlorides (Cl^-^) was determined after extraction of lettuce samples in 2% acetic acid (puriss. p.a., Avantor Performance Materials). The content of all ions was assessed using the AQ2 discrete analyzer (Seal Analytical, USA), using the analytical protocols attached to it (Smoleń et al., 2019).

### 2.5 Daily intake, percentage of recommended daily intake and risk quotient for iodine intake

Based on the results of determinations of iodine content in lettuce leaves, the daily iodine intake (DI-I) and the percentage of the recommended daily intake for iodine (% RDA-I) were calculated by consuming 50 g and 100 g portions of fresh lettuce leaves enriched with various iodine compounds. The RDA-I value recommended by the World Health Organization (WHO) based on the proposals of the United Nations Children’s Fund (UNICEF) and the International Council for the Control of Iodine Deficiency Disorders (ICCIDD) for adolescents over 12 years of age and adults was used for the calculations, amounting to 150 µg (WHO, 2022).

The health safety of potential consumers was also assessed on the basis of the hazard quotient (HQ), which determines the risk to human health resulting from the consumption of I contained in 50 g and 100 g portions of fresh lettuce leaves by adults weighing 70 kg. HQ-I values were calculated according to the mathematical formulas contained in the protocol of the United States Environmental Protection Agency (IRIS, 2011).

### 2.6 Statistical analysis

All determinations were performed in four replicates. The obtained results were statistically analyzed using the Statistica 13.1 program (StatSoft Inc., Tulsa, Oklahoma, USA). One-way analysis of variance (ANOVA) was used to analyze the data. Significance of differences between the mean values was assessed using Duncan’s *post hoc* test for a significance level of p ≤ 0.05.

## 3 Results

### 3.1 Weight of the whole lettuce and its head, dry matter content and basic chemical composition

**Table 1** presents the weight of the whole plant and its head, dry matter content and the basic chemical composition of lettuce biofortified with various iodine compounds. The use of potassium iodate and iodoquinolines for supplementation had a significant effect on increasing the weight of both the lettuce head and the whole plant. The average weight gain of the whole plant was 17.84%, while the average head weight gain was 17.03%. The highest weight of the head and the whole plant was recorded for lettuce enriched with 5-chloro-7-iodo-8-quinolinol, which increased by 26.48 and 26.94%, respectively, compared to the control. Biofortification with the tested forms of iodine did not cause significant changes in the content of dry matter in lettuce leaves. The exception was the use of 8-hydroxy-7-iodo-5-quinolinesulfonic acid, where its concentration was 0.56% higher than in the control sample. Lettuce supplementation with the tested iodine compounds resulted in a decrease in the content of protein, crude fat and ash. The average decrease in their concentration was 4.96, 16.88 and 4.22%, respectively. In plants enriched with 8-hydroxy-7-iodo-5-quinolinesulfonic acid, the lowest content of protein (decrease in concentration by 8.95%) and crude fat (decrease by 22.76%) was found, and in lettuce biofortified with KIO_3_ and 5,7-diiodo-8-quinolinol, the lowest ash concentration was observed (a decrease of 5.88 and 5.52%, respectively). Plants biofortified with the studied forms of iodine were characterized by a higher content of assimilable carbohydrates and dietary fiber (compared to non-supplemented plants). The average increase in their level was 2.92% and 8.90%, respectively. On the other hand, in the case of plants enriched with 4-hydroxy-8-iodo-3-quinolinecarboxylic acid, the concentration of available carbohydrates was lower by 3.96% than in the control. The highest content of available carbohydrates was recorded in lettuce treated with 5,7-diiodo-8-quinolinol. It was 8.85% higher than in the control lettuce.

**Table 1:** Weight, dry matter content and basic chemical composition of lettuce biofortified with various forms of iodine.

Treatment	Weight of the whole plant (head + root) [g]	Weight of the lettuce head [g]	Dry matter [% dry weight]	Protein [g 100 g^-1^ dry weight]	Crude fat [g 100 g^-1^ dry weight]	Digestible carbohydrates [g 100 g^-1^ dry weight]	Dietary fiber [g 100 g^-1^ dry weight]	Ash [g 100 g^-1^ dry weight]
Control	342.7 ± 6.0a	306.9 ± 5.9a	4.4 ± 0.2a	25.8 ± 0.4c	3.9 ± 0.1d	23.7 ± 0.5b	24.4 ± 0.4a	22.1 ± 0.2c
Potassium iodate	401.6 ± 12.4c	359.9 ± 11.3c	4.5 ± 0.2a	24.7 ± 0.3b	3.5 ± 0.1c	24.0 ± 0.4b	27.0 ± 0.2cd	20.8 ± 0.1a
8-hydroxy-7-iodo-5-quinolinesulfonic acid	379.7 ± 9.2b	329.8 ± 9.8b	5.0 ± 0.1b	23.5 ± 0.3a	3.0 ± 0.0a	24.9 ± 0.4c	27.2 ± 0.4d	21.3 ± 0.3b
5-chloro-7-iodo-8-quinolinol	433.5 ± 8.0e	389.6 ± 7.0e	4.4 ± 0.1a	24.6 ± 0.3b	3.3 ± 0.1b	24.6 ± 0.1c	26.2 ± 0.5c	21.3 ± 0.2b
5,7-diiodo-8-quinolinol	419.9 ± 6.9d	377.2 ± 5.6d	4.4 ± 0.0a	24.8 ± 0.4b	3.2 ± 0.1b	25.8 ± 0.3d	25.3 ± 0.2b	20.9 ± 0.2a
4-hydroxy-8-iodo-3-quinolinecarboxylic acid	384.5 ± 8.6b	339.4 ± 8.5b	4.4 ± 0.2a	25.1 ± 0.2b	3.3 ± 0.1b	22.8 ± 0.5a	27.2 ± 0.2d	21.6 ± 0.2b

The results are presented as mean ± standard deviation (n=4). Values followed by the same letters are not significantly different at p < 0.05.

### 3.2 The content of iodine, iodoquinolines, iodide ions, micro- and macroelements

When compared to control the concentration of iodine in the lettuce plants increased on average 70.26 times, and its level ranged from 8.99 mg I·kg^-1^ dry weight (after 5-chloro-7-iodo-8-quinolinol application) to 117.50 mg I·kg^-1^ dry weight (after 8-hydroxy-7-iodo-5-quinolinesulfonic acid application) (**Table 2**).

**Table 2:** Concentration of minerals in lettuce biofortified with various forms of iodine.

Treatment	Macroelements [% dry weight]						
	N	P	S	Na	K	Mg	Ca
Control	4.3 ± 0.1b	1.1 ± 0.0e	0.3 ± 0.0a	0.1 ± 0.0a	10.2 ± 0.1b	0.4 ± 0.0a	1.4 ± 0.1a
Potassium iodate	3.9 ± 0.0a	0.9 ± 0.0a	0.3 ± 0.0a	0.2 ± 0.0cd	11.4 ± 0.1d	0.5 ± 0.0c	1.9 ± 0.1c
8-hydroxy-7-iodo-5-quinolinesulfonic acid	4.2 ± 0.2b	1.0 ± 0.0c	0.3 ± 0.0a	0.2 ± 0.0cd	8.9 ± 0.2a	0.4 ± 0.0a	1.6 ± 0.1b
5-chloro-7-iodo-8-quinolinol	4.3 ± 0.1b	0.9 ± 0.0b	0.3 ± 0.0a	0.1 ± 0.0bc	10.3 ± 0.3b	0.5 ± 0.0c	1.6 ± 0.1b
5,7-diiodo-8-quinolinol	4.0 ± 0.2a	1.0 ± 0.0cd	0.3 ± 0.0a	0.2 ± 0.0d	10.8 ± 0.4c	0.5 ± 0.0c	1.9 ± 0.0c
4-hydroxy-8-iodo-3-quinolinecarboxylic acid	4.2 ± 0.1b	1.0 ± 0.0d	0.3 ± 0.0a	0.1 ± 0.0ab	10.0 ± 0.5b	0.4 ± 0.0b	1.6 ± 0.1b
Treatment	Microelements [mg kg^-1^ dry weight]						
	I	Fe	Cu	Zn	Mn	Mo	B
Control	0.5 ± 0.1a	165.4 ± 4.0a	12.0 ± 0.7d	77.2 ± 7.9b	90.8 ± 4.6b	1.1 ± 0.0b	46.9 ± 3.6a
Potassium iodate	19.9 ± 1.1c	168.9 ± 7.6ab	9.8 ± 0.4c	70.4 ± 0.9b	99.2 ± 2.9b	1.2 ± 0.0c	67.5 ± 2.7c
8-hydroxy-7-iodo-5-quinolinesulfonic acid	117.5 ± 7.5e	171.6 ± 3.5ab	5.7 ± 0.5a	54.9 ± 6.9a	75.5 ± 6.0a	0.7 ± 0.0a	66.4 ± 3.4c
5-chloro-7-iodo-8-quinolinol	9.0 ± 1.3b	177.4 ± 4.6b	8.8 ± 0.3bc	100.9 ± 4.5c	179.0 ± 5.2c	1.4 ± 0.2d	63.1 ± 2.3bc
5,7-diiodo-8-quinolinol	26.7 ± 2.5d	191.9 ± 8.9c	8.2 ± 0.6b	109.2 ± 3.1d	215.0 ± 9.6e	1.4 ± 0.1d	71.8 ± 1.7d
4-hydroxy-8-iodo-3-quinolinecarboxylic acid	13.1 ± 0.9b	174.9 ± 4.5b	13.3 ± 1.3e	98.0 ± 4.2c	205.7 ± 6.7d	1.3 ± 0.1c	59.5 ± 2.9b

The results are presented as mean ± standard deviation (n=4). Values followed by the same letters are not significantly different at p < 0.05.

Plants fortified with 8-hydroxy-7-iodo-5-quinolinesulfonic acid were characterized by the highest concentration of this element, which was 221.7 times higher than in control plants (5.9 times higher compared to the sample treated with KIO_3_). In treatments with testing of individual iodoquinolines, there was a significant increase in their content as well as quinoline in roots, leaves and RootSec, respectively (**Table 3**). After application of all tested iodine compounds (including KIO_3_), a significant increase in the content of iodide ions (I^-^) in roots and leaves was found − they were not determined in RootSec.

**Table 3:** Concentrations of iodides (I^-^), quinoline, 8-hydroxy-7-iodo-5-quinolinesulfonic acid, 5-chloro-7-iodo-8-quinolinol, 5,7-diiodo-8-quinolinol and 4-hydroxy-8-iodo-3-quinolinecarboxylic acid in leaves and roots of lettuce as well as root secretions (RootSec).

Part of plant/RootSec*	Treatment	Iodides (I^-^) [µg kg^-1^ dry weight]	Quinoline [µg kg^-1^ dry weight]	8-hydroxy-7-iodo-5-quinolinesulfonic acid [µg kg^-1^ dry weight]	5-chloro-7-iodo-8-quinolinol [µg kg^-1^ dry weight]	5,7-diiodo-8-quinolinol [µg kg^-1^ dry weight]	4-hydroxy-8-iodo-3-quinolinecarboxylic acid [µg kg^-1^ dry weight]
			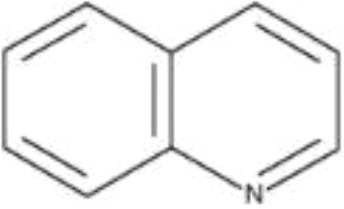	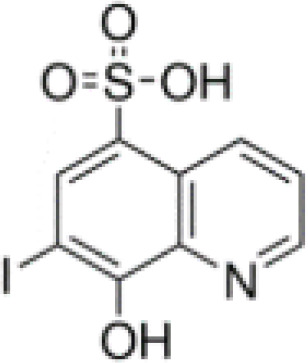	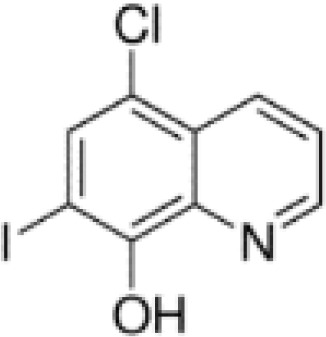	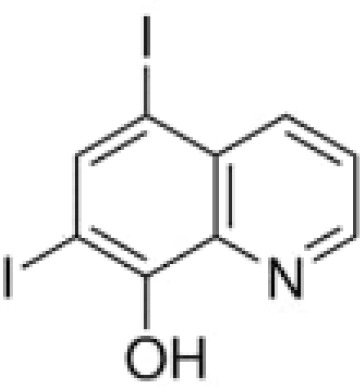	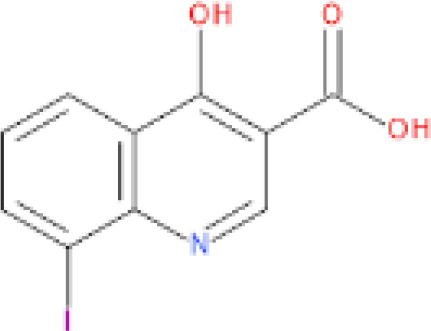
		Leaves					
Leaves	Control	217.3 ± 16.2a	1.9 ± 0.2a	0.0 ± 0.0a	4.4 ± 0.5a	0.0 ± 0.0a	0.0 ± 0.0a
	Potassium iodate	7288.8 ± 807.8b	5.6 ± 0.8b	0.6 ± 0.1b	1.9 ± 0.1a	0.0 ± 0.0a	0.0 ± 0.0a
	8-hydroxy-7-iodo-5-quinolinesulfonic acid	67218.3 ± 8506.4c	5.7 ± 1.6b	9.7 ± 0.3d	2.2 ± 0.2a	0.0 ± 0.0a	0.0 ± 0.0a
	5-chloro-7-iodo-8-quinolinol	4440.7 ± 296.2ab	11.3 ± 2.0d	1.1 ± 0.2c	469.8 ± 32.9b	0.0 ± 0.0a	77.6 ± 18.9b
	5,7-diiodo-8-quinolinol	8088.5 ± 1018.4b	8.7 ± 1.4c	0.5 ± 0.1b	16.2 ± 2.0a	195.7 ± 65.8a	84.3 ± 8.9b
	4-hydroxy-8-iodo-3-quinolinecarboxylic acid	6143.6 ± 1929.5b	10.7 ± 1.6cd	1.3 ± 0.2c	4.8 ± 1.9a	2574.4 ± 452.9b	588.2 ± 14.7c
		Roots					
Roots	Control	154.0 ± 75.9a	44.6 ± 6.6a	1.2 ± 0.2a	40.9 ± 0.5a	42.8 ± 3.6a	0.0 ± 0.0a
	Potassium iodate	4548.3 ± 1855.8a	48.9 ± 2.4ab	2.2 ± 0.3a	22.3 ± 6.4a	18.5 ± 4.5a	9.6 ± 1.4a
	8-hydroxy-7-iodo-5-quinolinesulfonic acid	44945.0 ± 1521.4b	70.1 ± 6.6c	41013.6 ± 4607.4b	9.6 ± 1.5a	242.9 ± 40.4a	0.0 ± 0.0a
	5-chloro-7-iodo-8-quinolinol	120367.4 ± 16456.3d	49.0 ± 2.3ab	2.0 ± 0.3a	21569.8 ± 2515.3b	193.1 ± 9.0a	0.0 ± 0.0a
	5,7-diiodo-8-quinolinol	65854.9 ± 5407.0c	54.2 ± 4.7b	0.8 ± 0.1a	163.3 ± 34.7a	27651.9 ± 2718.3b	0.0 ± 0.0a
	4-hydroxy-8-iodo-3-quinolinecarboxylic acid	7767.4 ± 1190.9a	48.5 ± 3.3ab	1.2 ± 0.2a	32.6 ± 5.0a	371.3 ± 86.2a	100687.2 ± 5368.9b
		RootSec*					
		Iodides (I^-^) (µg·dm^-3^)	Quinoline (µg·dm^-3^)	8-hydroxy-7-iodo-5-quinolinesulfonic acid (µg·dm^-3^)	5-chloro-7-iodo-8-quinolinol (µg·dm^-3^)	5,7-diiodo-8-quinolinol (µg·dm^-3^)	4-hydroxy-8-iodo-3-quinolinecarboxylic acid (µg·dm^-3^)
RootSec	Control	no data**	1264.2 ± 29.8b	0.0 ± 0,0a	2.9 ± 0.2a	1.2 ± 0.3a	2760.1 ± 562.6c
	Potassium iodate	no data	3406.2 ± 20.6e	0.0 ± 0.0a	1.7 ± 1.1a	0.0 ± 0.0a	3052.7 ± 613.4c
	8-hydroxy-7-iodo-5-quinolinesulfonic acid	no data	3530.6 ± 428.3e	0.2 ± 0.0b	3.2 ± 0.9a	9.6 ± 1.7b	843.1 ± 122.9a
	5-chloro-7-iodo-8-quinolinol	no data	1787.5 ± 35.9c	0.0 ± 0.0a	773.0 ± 13.5b	2.4 ± 0.1a	1783.6 ± 93.3b
	5,7-diiodo-8-quinolinol	no data	2527.5 ± 165.0d	0.0 ± 0.0a	7.0 ± 2.6a	148.2 ± 10.2c	1549.2 ± 168.9b
	4-hydroxy-8-iodo-3-quinolinecarboxylic acid	no data	490.6 ± 28.9a	0.0 ± 0.0a	0.0 ± 0.0a	0.0 ± 0.0a	2915.8 ± 418.8c

*RootSec - Results of the determination of individual compounds in secretions collected as a result of root pressure – this is in white secretion on the surface of the root neck after cutting the heads (lettuce leaves). **the analysis was not performed due to the too small amount of sample, which was intended only for the analysis of other exchangeable compounds in this table using the LC-MS/MS technique.

Results “0.0 ± 0.0” means below limit of quantification (LOQ) exactly according method described by Smoleń et al. (Smoleń et al. 2021; Smoleń et al., 2023) and Krzemińska et al. (2023) for determination iodine-organic compounds by LC-MS/MS method. Means in the column followed by different letters differ significantly at p < 0.05 (n=4).

Biofortification with various forms of iodine significantly affected the concentration of most macro- and microelements in lettuce leaves (**Table 2**). An average of 0.11% reduction in P content was observed (relative to control plants). The lowest level of P was recorded for lettuce treated with KIO_3_, and the decrease in its content was 0.21%. The use of the tested forms of iodine did not contribute to significant changes in the content of S. On the other hand, in the samples supplemented with KIO_3_ and 5,7-diiodo-8-quinolinol, the N concentration was 0.42% and 0.30% lower, respectively (compared to non-biofortified lettuce). Plant enrichment with iodine compounds also resulted in higher levels of Na, Mg and Ca, on average by 0.05, 0.08 and 0.28%. Nevertheless, the increase in Na concentration in lettuce treated with 4-hydroxy-8-iodo-3-quinolinecarboxylic acid was not statistically significant. In addition, it was observed that in plants supplemented with 8-hydroxy-7-iodo-5-quinolinesulfonic acid, the Mg content was similar to its concentration in the control sample. In lettuce biofortified with KIO_3_ and 5,7-diiodo-8-quinolinol, the highest concentrations of Ca were recorded, which were 0.44% and 0.46% higher, respectively, than in unenriched lettuce. The tested iodine compounds influenced the content of K in lettuce in various ways. Compared to control plants, samples fortified with 8-hydroxy-7-iodo-5-quinolinesulfonic acid were characterized by 1.29% lower concentration of this element, while plants supplemented with KIO_3_ and 5,7-diiodo-8-quinolinol - higher, respectively 1.22 and 0.61%.

Lettuce supplementation with various forms of iodine contributed to an increase in the levels of Fe, Zn, Mn, Mo and B (on average by 9.65, 32.98, 120.24, 30.24 and 40.10%, respectively; relative to untreated lettuce) (**Table 2**). On the other hand, the use of KIO_3_ and 8-hydroxy-7-iodo-5-quinolinesulfonic acid did not cause statistically significant changes in Fe content. Moreover, in the case of biofortification with KIO_3_, the level of Zn and Mn was similar to their concentration in unenriched control lettuce. In lettuce biofortified with 8-hydroxy-7-iodo-5-quinolinesulfonic acid, the lowest concentrations of Zn, Mn and Mo were found, and it was the only research sample with lower levels of these elements compared to the control sample (decrease in content by respectively 28.97, 16.82 and 36.19%). Lettuce supplemented with 5,7-diiodo-8-quinolinol was characterized by the highest levels of Fe, Zn, Mn, Mo and B (concentration increase by 16.00, 41.36, 136.84, 38.10 and 53.11%, respectively). In lettuce fortified with 5-chloro-7-iodo-8-quinolinol, the highest concentration of Mo was also found, and the increase in its content was at the same level as in the case of lettuce supplemented with 5,7-diiodo-8-quinolinol. Enrichment of lettuce with various forms of iodine resulted in a decrease in Cu level, by an average of 32.57%. However, in the case of plants treated with 4-hydroxy-8-iodo-3-quinolinecarboxylic acid, the Cu concentration was higher by 10.65%. The lowest content of Cu was found in plants supplemented with 8-hydroxy-7-iodo-5-quinolinesulfonic acid, where the decrease in its concentration was 52.50%.

### 3.3 The content of water-soluble vitamins

Biofortification of lettuce with the use of the tested iodine compounds had a significant impact on the content of some vitamins from group B vitamins (**Table 4**). In the research, an increase in the concentration of vitamin B1, B2 and B5 in lettuce was found on average by 25.57, 7.33 and 25.80%, respectively (relative to the control). Nevertheless, in plants enriched with 5,7-diiodo-8-quinolinol, the content of vitamin B1 and B5 was statistically insignificant, while the application of 5-chloro-7-iodo-8-quinolinol did not contribute to significant changes in the concentration of vitamin B2 *versus* control. In the case of plants supplemented with an inorganic form of iodine (KIO_3_), a decrease in the content of vitamin B1 and B5 was recorded by 10.69 and 11.06%. The highest concentration of vitamin B1 and B5 was observed in lettuce biofortified with 8-hydroxy-7-iodo-5-quinolinesulfonic acid, where *versus* control the increase in their level was 41.38 and 43.13%, respectively. Lettuce treated with various forms of iodine was also characterized by an average of 22.92% lower levels of vitamin PP. The lowest concentration of vitamin PP was found in lettuce fortified with KIO_3_ and 4-hydroxy-8-iodo-3-quinolinecarboxylic acid, and the average decrease in its level for both research samples was 31.25%. No significant changes in the content of vitamin B9 were observed between the tested iodine combination and the control sample. In plants supplemented with 8-hydroxy-7-iodo-5-quinolinesulfonic acid and 4-hydroxy-8-iodo-3-quinolinecarboxylic acid, the concentration of vitamin B3 was 4.86 and 1.94 times higher, respectively, than in non-biofortified plants. The use of the tested iodine compounds had a different effect on the level of vitamin B6. In plants enriched with KIO_3_ and 5,7-diiodo-8-quinolinol, there was a 21.15 and 19.23% decrease in the concentration of vitamin B6 (compared to the control), and in plants supplemented with 5-chloro-7-iodo-8-quinolinol, a 23.08% increase in its level was found. In turn, in lettuce treated with 8-hydroxy-7-iodo-5-quinolinesulfonic acid and 4-hydroxy-8-iodo-3-quinolinecarboxylic acid, the content of vitamin B6 was comparable to its concentration in control plants.

**Table 4:** Content of water-soluble vitamins in lettuce biofortified with various forms of iodine.

Treatment	Vitamin B1 (thiamine hydrochloride) [mg kg^-1^ dry weight]	Vitamin B2 (riboflavin) [mg kg^-1^ dry weight]	Vitamin B3 (nicotinic acid) [mg kg^-1^ dry weight]	Vitamin PP (nicotinamide) [mg kg^-1^ dry weight]	Vitamin B5 (pantothenic acid) [mg kg^-1^ dry weight]	Vitamin B6 (pyridoxine hydrochloride) [mg kg-^1^ dry weight]	Vitamin B9 (folic acid) [mg kg^-1^ dry weight]	Vitamin C [mg 100 g^-1^ fresh weight]	
								L-ascorbic acid	Dehydroascorbic acid
Control	5.8 ± 0.0b	9.3 ± 0.3a	0.5 ± 0.1a	0.5 ± 0.0c	6.3 ± 0.2b	0.5 ± 0.0b	0.3 ± 0.0a	10.3 ± 0.3b	0.9 ± 0.2a
Potassium iodate	5.2 ± 0.2a	10.1 ± 0.2c	0.5 ± 0.1a	0.3 ± 0.0a	5.6 ± 0.2a	0.4 ± 0.1a	0.3 ± 0.0a	10.9 ± 0.2c	1.1 ± 0.1a
8-hydroxy-7-iodo-5-quinolinesulfonic acid	8.2 ± 0.2e	10.3 ± 0.1c	2.4 ± 0.2c	0.4 ± 0.0ab	9.1 ± 0.5d	0.5 ± 0.1b	0.2 ± 0.0a	13.5 ± 0.6e	0.9 ± 0.1a
5-chloro-7-iodo-8-quinolinol	6.6 ± 0.1c	9.2 ± 0.2a	0.4 ± 0.1a	0.4 ± 0.0b	7.6 ± 0.4c	0.6 ± 0.0c	0.2 ± 0.0a	10.0 ± 0.1b	1.6 ± 0.1b
5,7-diiodo-8-quinolinol	6.2 ± 0.1b	9.6 ± 0.1b	0.3 ± 0.1a	0.4 ± 0.1b	6.7 ± 0.4b	0.4 ± 0.0a	0.2 ± 0.0a	12.8 ± 0.2d	2.3 ± 0.2c
4-hydroxy-8-iodo-3-quinolinecarboxylic acid	7.0 ± 0.6d	10.1 ± 0.0c	1.0 ± 0.1b	0.3 ± 0.0a	7.2 ± 0.3c	0.6 ± 0.0b	0.3 ± 0.0a	8.7 ± 0.5a	1.4 ± 0.2b

The results are presented as mean ± standard deviation (n=4). Values followed by the same letters are not significantly different at p < 0.05.

The highest content of ascorbic acid (AA) and dehydroascorbic acid (DHA) was found in plants enriched with 8-hydroxy-7-iodo-5-quinolinesulfonic acid and 5,7-diiodo-8-quinolinol, respectively, and their concentration was 1.32 and 2.55 times higher than in the control plants (**Table 4**). Lettuce fortified with 4-hydroxy-8-iodo-3-quinolinecarboxylic acid showed a 15.68% decrease in AA level. Moreover, no significant changes in AA concentration in lettuce supplemented with 5-chloro-7-iodo-8-quinolinol and DHA content in plants after application of KIO_3_ and 8-hydroxy-7-iodo-5-quinolinesulfonic acid were found (compared to control lettuce).

### 3.4 Total polyphenol content and antioxidant activity

The use of almost all tested iodine compounds resulted in an average 1.69-fold increase in total polyphenol content and an average 1.32-fold increase in antioxidant activity in lettuce (**Table 5**). In plants enriched with 5-chloro-7-iodo-8-quinolinol and 4-hydroxy-8-iodo-3-quinolinecarboxylic acid, there was a decrease in antioxidant activity by 23.75 and 35.19%, respectively (compared to the control). In lettuce treated with 8-hydroxy-7-iodo-5-quinolinesulfonic acid, the highest concentration of total polyphenols was observed, which was 2.33 times higher than in the control sample. The highest antioxidant activity was found in lettuce biofortified with 5,7-diiodo-8-quinolinol, where its 1.43-fold increase was found.

**Table 5:** Total polyphenol content and antioxidant activity of lettuce biofortified with various forms of iodine.

Treatment	Total polyphenol content [mg 100 g^-1^ dry weight]	Antioxidant activity [µmol Trolox g^-1^ dry weight]
Control	22.9 ± 0.5a	64.3 ± 1.3c
Potassium iodate	30.0 ± 0.2c	72.7 ± 2.4d
8-hydroxy-7-iodo-5-quinolinesulfonic acid	53.2 ± 0.1e	89.0 ± 1.6e
5-chloro-7-iodo-8-quinolinol	29.9 ± 0.1c	49.0 ± 1.8b
5,7-diiodo-8-quinolinol	51.6 ± 0.3d	92.0 ± 0.7f
4-hydroxy-8-iodo-3-quinolinecarboxylic acid	28.7 ± 0.1b	41.6 ± 0.6a

The results are presented as mean ± standard deviation (n=4). Values followed by the same letters are not significantly different at p < 0.05.

### 3.5 The content of nitrogen compounds and chlorides

The use of the tested forms of iodine contributed to an average 29.31% increase in the concentration of ammonium ions (NH_4_^+^) in lettuce leaves (compared to the control sample) (**Table 6**). The lowest content of nitrate (III) (NO_2_^-^) and nitrate (V) (NO_3_^-^) ions was recorded in lettuce biofortified with 8-hydroxy-7-iodo-5-quinolinesulfonic acid, and their level was 6.33 and 1.44 times lower, respectively, than in control lettuce. In plants supplemented with KIO_3_, no significant changes in the concentration of NO_2_^-^ ions were observed (compared to unenriched plants). The lowest concentration of chloride ions (Cl^-^) was found in lettuce treated with 5-chloro-7-iodo-8-quinolinol and 5,7-diiodo-8-quinolinol, and it was lower by 40.82 and 36.63%, respectively, compared to the control.

**Table 6:** Concentration of nitrogen compounds and chlorides in lettuce biofortified with various forms of iodine.

Treatment	NO_3_^-^ [mg kg^-1^ fresh weight]	NO_2_^-^ [mg kg^-1^ fresh weight]	NH_4_^+^ [mg kg^-1^ fresh weight]	Cl^-^ [mg kg^-1^ fresh weight]
Control	4122.2 ± 267.2d	2.7 ± 0.2d	17.0 ± 0.9a	216.0 ± 2.2d
Potassium iodate	3818.6 ± 132.5c	2.9 ± 0.1d	24.3 ± 1.1c	170.2 ± 11.9b
8-hydroxy-7-iodo-5-quinolinesulfonic acid	2854.3 ± 230.0a	0.4 ± 0.0a	18.8 ± 0.1b	198.1 ± 7.5c
5-chloro-7-iodo-8-quinolinol	3472.8 ± 168.9b	0.6 ± 0.0ab	24.3 ± 1.3c	127.8 ± 4.3a
5,7-diiodo-8-quinolinol	3506.3 ± 59.9b	1.1 ± 0.2c	23.1 ± 0.6c	136.9 ± 4.4a
4-hydroxy-8-iodo-3-quinolinecarboxylic acid	3675.9 ± 82.2bc	0.8 ± 0.1b	19.7 ± 0.1b	204.6 ± 8.6c

The results are presented as mean ± standard deviation (n=4). Values followed by the same letters are not significantly different at p < 0.05.

### 3.6 Daily intake, percentage of recommended daily intake and hazard quotient for iodine intake

The use of the studied forms of iodine for the biofortification of lettuce resulted in an increase in the daily intake of iodine, and thus also an increase in the percentage of its recommended daily intake and the HQ-I for iodine intake (**Table 7**). In lettuce treated with 8-hydroxy-7-iodo-5-quinolinesulfonic acid, the highest values of the adopted indicators of health safety assessment of potential consumers (DI-I, % RDA-I, HQ-I) were found. These values were significantly different from the results obtained in other treatments. The average daily iodine intake ranged from 19.9 to 293.2 µg I/day (13.27-195.46% RDA-I) and from 39.8 to 586.39 µg I/day (26.53-390.93% RDA-I) for 50 and 100 g servings of fresh lettuce leaves, respectively supplemented with the tested forms of iodine. The obtained HQ values for the intake of iodine contained in the enriched lettuce were in all tested treatments lower than 1.0 (a value of 1.0 is considered harmful and/or toxic to humans). They ranged from 0.01 to 0.19 and from 0.02 to 0.38 for HQ-I 50g and HQ-I 100g portions, respectively.

**Table 7:** Daily intake of iodine (DI-I), percentage of recommended daily allowance for iodine (% RDA-I) and hazard quotient for iodine intake (HQ-I) through consumption of 50 and 100 g portions of fresh lettuce leaves by adults 70 kg body weight.

Treatment	DI-I with a 50g of lettuce [mg I day^-1^]	DI-I with a 100g of lettuce [mg I day^-1^]	% RDA-I (in a 50g portion of lettuce)	% RDA-I (in a 100g portion of lettuce)	HQ-I for a 50g portion of lettuce	HQ-I for a 100g portion of lettuce
Control	0.001 ± 0.00a	0.002 ± 0.001a	0.79 ± 0.19a	1.58 ± 0.37a	0.001 ± 0.00a	0.001 ± 0.00a
Potassium iodate	0.04 ± 0.002ab	0.09 ± 0.005ab	29.64 ± 1.57ab	59.29 ± 3.14ab	0.03 ± 0.002a	0.05 ± 0.005a
8-hydroxy-7-iodo-5-quinolinesulfonic acid	0.29 ± 0.08c	0.59 ± 0.15c	195.46 ± 51.55c	390.93 ± 103.11c	0.19 ± 0.05b	0.38 ± 0.11b
5-chloro-7-iodo-8-quinolinol	0.02 ± 0.003ab	0.04 ± 0.01ab	13.27 ± 1.96ab	26.53 ± 3.92ab	0.01 ± 0.002a	0.02 ± 0.004a
5,7-diiodo-8-quinolinol	0.06 ± 0.01b	0.12 ± 0.02b	39.20 ± 5.64b	78.40 ± 11.28b	0.03 ± 0.005a	0.07 ± 0.01a
4-hydroxy-8-iodo-3-quinolinecarboxylic acid	0.03 ± 0.002ab	0.06 ± 0.004ab	19.32 ± 1.26ab	38.64 ± 2.53ab	0.02 ± 0.001a	0.03 ± 0.002a

The results are presented as mean ± standard deviation (n=4). Values followed by the same letters are not significantly different at p < 0.05.

## 4 Discussion

The feasibility of biofortification depends, among others, on ensuring high yields and profitability for producers (Freire et al., 2020). Research conducted by Voogt et al. (2010) showed that the supplementation of lettuce with iodine in the form of iodide (I^-^) and iodate (IO_3_^-^) in various concentrations, both in winter and summer, did not affect the weight of its head and root (biomass production). In turn, Sularz et al. (2020) observed that the use of 5-iodosalicylic acid and 3,5-diiodosalicylic acid (organic compounds) contributed to a significant (about 2-fold) reduction in the weight of the head of lettuce. The results obtained by our team proved, however, that the biofortification of lettuce with iodoquinolines and KIO_3_ resulted in an increase in the weight of the whole plant and its head. This increase was particularly evident in the case of lettuce enriched with 5-chloro-7-iodo-8-quinolinol, where the highest weight of the whole plant and head of lettuce was recorded. The growth and yield of these plants can be largely determined by the dose and type of compound used for biofortification, appropriate water content, agrotechnical treatments, cultivation method and environmental conditions (light and temperature) (Wu et al., 2019). Therefore, the potential use of iodoquinolines to enrich lettuce could be promising from the farmers’ point of view, due to obtaining higher yields than by fortification of this plant with other forms of iodine.

Dry matter is an important parameter determining food quality, technological suitability, and even durability and resistance to transport (Borreani et al., 2018). Gonnella et al. (2019) found that biofortification with iodine (with KIO_3_) of four *Brassica* genotypes (broccoli raab, curly kale, mizuna, red mustard) did not significantly affect the dry matter content. Similar observations were reported in studies where the biological quality of seedlings of two varieties of radish enriched with iodine (in the form of KI) was assessed (Krzepiłko et al., 2021). Our research also did not show significant changes in the content of dry matter in almost all research samples, and the increase in its level in lettuce enriched with 8-hydroxy-7-iodo-5-quinolinesulfonic acid was probably due to, among others, from the chemical form of iodine used for supplementation.

The basic chemical composition is one of the factors determining the nutritional value of food. Sularz et al. (2020) have observed that supplementation of lettuce with various forms of iodine (KIO_3_, 5-ISA, 3.5-diISA) does not affect the content of assimilable carbohydrates, dietary fiber and ash, while the use of iodosalicylic acids causes a significant decrease in protein level. In samples enriched with KIO_3_, they also noted an increase in crude fat concentration. The results obtained by our team showed that the use of iodoquinolines and KIO_3_ for lettuce biofortification contributes to the reduction of protein, crude fat and ash content. In almost all treatments, we found a significant increase in the level of available carbohydrates and dietary fiber (in contrast to the control sample). The results of our research do not allow for a clear interpretation of these results in terms of the course of physiological processes in plants. The increased content of available carbohydrates is probably due to their enhanced remobilization from storage sugars (Blasco et al., 2011b; Klopotek and Kläring, 2014). In addition, changes in the basic chemical composition of these plants may be determined by the form of iodine used for supplementation, as well as mutual interactions between iodine and other elements at the level of their cultivation and growth phase. Certain minerals play a structural and regulatory role in relation to the metabolism of carbohydrates, amino acids and lipids in plants (Chen et al., 2021).

The recent publications indicate that 5-iodosalicylic acid (5-ISA), 3,5-diiodosalicylic acid (3,5-diISA), 2-iodobenzoic acid (2-IBeA) and 4-iodobenzoic acid (4-IBeA) can be not only a source of iodine for plants, but also can modify their chemical composition in a different way than inorganic iodine (Halka et al., 2019). Exogenous application of these compounds appears to presumably increase the ability of plants to synthesize organic iodine compounds containing iodine bound to an aromatic group (Halka et al., 2020). Smoleń et al. (2022) showed that the use of 5-ISA results in the highest levels of iodine accumulation and uptake in lettuce grown in both peat and mineral soil. In the study Krzemińska et al. (2023) was found that 8-hydroxy-7-iodo-5-quinolinesulfonic acid and 5-chloro-7-iodo-8-quinolinol was taken up by potato plants. These iodoquinolines were accumulated in tubers. However, the total iodine content in the tubers after their application was approximately half lower than after KIO_3_ application. In our study, the highest content of iodine was found in lettuce enriched with 8-hydroxy-7-iodo-5-quinolinesulfonic acid, and a higher level of iodine than in plants supplemented with KIO_3_ was also found in lettuce treated with 5,7-diiodo-8-quinolinol. The explanation why the highest iodine concentration was observed in plants supplemented with 8-hydroxy-7-iodo-5-quinolinesulfonic acid may be that in the presence of a highly polar sulfone group (-SO_3_H), hydrophobic aromatic compounds become hydrophilic and acidic, which increases their solubility in the water (Nandhakumar et al., 2022). It is assumed that the forms of microelements that dissolve in water and soil solutions are the easiest to be absorbed by plants (Masunaga and Fong, 2018). Moreover, the amount of iodine contained in plants enriched with iodoquinolines is probably affected by the number and distribution of substituents (including primarily iodine substituents) at the aromatic rings and their distance from the nitrogen atom incorporated in the aromatic ring. However, the current state of knowledge does not allow for the verification of the above-mentioned assumptions. In addition, the process of biofortification is not simple and obvious, and the uptake of iodine organic compounds as iodoquinolines by roots from the nutrient solution (or soil) is hindered by e.g. particle size of these compounds (compared to IO_3_^-^) and the multi-phase transformation processes that this element undergoes in the rhizosphere environment and inside root cells. The accumulation of iodine (as I^-^ ions) in leaves depends mainly on its transport through the xylem. I^-^ uptake is catalyzed by H^+^/anion symporters and then released into the xylem via anion channels (Buturi et al., 2021). Our results indicate that after application of individual iodoquinolines, there was a significant increase in their content in roots and leaves and in RootSec. We also observed an increase in the content of I^-^ ions in roots and leaves after the application of all iodoquinolines. These results can be interpreted as follows: iodoquinolines are transported from roots to leaves and undergo catabolism processes, which leads to among others the formation of inorganic I^-^ ions. Iodoquinolines and product of its metabolism (I^-^ ions) are transported from roots to the leaves. Moreover, the catabolism of exogenous iodoquinolines to I^-^ ions can also place in the leaves. In the study Smoleń et al. (2023) in plants treated with iodineorganic compounds as iodosalicylates the synthesis of endogenous iodoquinolines was observed – the iodoquinolines was indicated in the exudates collected as a result of root pressure (RootSec) of plants treated with iodine compounds or salicylic acid (compared to the control). In conclusion, the results of our research and Smoleń et al. (2023) do not allow to indicate a clear mechanism for the transport of iodoquinolines from roots to leaves. The transport of these organoiodine compounds presumably occurs via an apoplastic and/or symplastic pathway or may be passive along with water transport in xylem.

Mineral components can perform building and physiological functions in plants. Physiological functions testify to their participation in many physiological processes of fundamental importance for plants, such as photosynthesis, respiration, hormonal balance, water management, nitrogen metabolism and metabolism of organic compounds (Hänsch and Mendel, 2009; de Bang et al., 2021). Biofortification with iodine contributes to changes in the assimilability of mineral compounds in plants. Krzepiłko et al. (2016) noted that the content of K in lettuce seedlings increased with the applied KI doses, the level of Ca, Zn, Fe and Cu did not change significantly, while the concentration of Na, Mg and Mn decreased as a result of biofortification. In turn, in another experiment, it was shown that the enrichment of lettuce with various forms of iodine increases the concentration of macro- and microelements, such as Ca, Mg, Na, Fe, Mo and B (Sularz et al., 2020). Our research has shown that the use of 5,7-diiodo-8-quinolinol caused the highest increasing the content of Na, Mg, Ca, Fe, Zn, Mn, B and Mo in lettuce. Sularz et al. (2020) showed that biofortification of lettuce with iodosalicylic acids and KIO_3_ results in lower Cu (and tendency to decreasing P) concentration in lettuce. Our results confirmed the observations of these authors, and also showed a decrease in the level of Cu and P in plants after iodine compounds application. Changes in the content of minerals in lettuce suggest that biofortification results in numerous interactions between elements. The concentration of minerals in the plant is usually positively correlated with their concentration in the root environment and they can influence the uptake of other elements in many ways (Islam et al., 2007; Buturi et al., 2021). In lettuce supplemented with 8-hydroxy-7-iodo-5-quinolinesulfonic acid, we observed strong antagonistic interactions between this compound *versus* Cu, Zn, Mn and Mo concentration in plants – however, without negative effects on yield. This may be explained by the fact that content of Cu, Zn, Mn and Mo may be in optimal amounts in relation to the nutritional requirements of plants. In turn, in plants enriched with 5,7-diiodo-8-quinolinol synergistic interactions between this compound *versus* Zn, Mn and B where we found − an approximately 1.5-2-fold increase in the content of these elements. It is worth mentioning that the total concentration of iodine in lettuce after application of 5,7-diiodo-8-quinolinol was more than 4 times lower than in lettuce, in which strong antagonistic reactions 8-hydroxy-7-iodo-5-quinolinesulfonic acid versus Cu, Zn, Mn and Mo were noted.

Plants are the source of most water-soluble vitamins, which are essential for both human metabolism and plants due to their participation in redox reactions and their role as enzyme cofactors (Asensi-Fabado and Munne-Bosch, 2010). At the moment, there are few publications evaluating the effect of biofortification with iodine on the content of B vitamins. Sularz et al. (2021) noted an increase in the concentration of vitamin B3 in lettuce fertilized with KIO_3_ and 3,5-diISA, and an increase in the level of vitamin B5 and PP in lettuce enriched with KIO_3_. In addition, they showed that the use of iodosalicylic acids for supplementation of these plants does not contribute to changes in the content of vitamin B1, B2, B5 and PP and causes a decrease in the level of vitamin B6 and B9. In our study we found that lettuce enriched with iodoquinolines is characterized by a higher concentration of vitamins B1, B2 and B5. In lettuce supplemented with KIO_3_, we also noted an increase in the level of vitamin B2. Moreover, biofortification with various forms of iodine did not significantly affect the level of vitamin B9 in lettuce. We only recorded a decrease in the concentration of vitamin PP after all tested iodine compounds. An increasing concentration of some B vitamins may be beneficial for plants, mainly due to their enhanced tolerance to stress (Titcomb and Tanumihardjo, 2019). Regarding the role of these vitamins in plant metabolism and health, thiamine (vitamin B1) has received the most attention. This vitamin is involved in key metabolic processes in plants, including carbon fixation (photosynthesis) and respiration (TCA cycle), and thanks to many coordinated reactions throughout their central metabolism, it can contribute to increased yields (Fitzpatrick and Chapman, 2020). Thiamine is also involved in the abiotic and biotic responses of plants to stress, and stress conditions cause an increase in its biosynthesis. Considering abiotic stress, it has been shown that salt, temperature, osmotic and oxidative stress can increase the expression of genes responsible for thiamine biosynthesis in plants (Hanson et al., 2016). Increased expression of these genes under stress is thought to provide more thiamine diphosphate (TDP; thiamine is its precursor) to provide metabolic enzymes dependent on it as a coenzyme, which in turn may support abiotic defense responses (Fitzpatrick and Chapman, 2020). Riboflavin (vitamin B2) and pantothenic acid (vitamin B5) are equally essential and likely have similar functions in the central metabolism of plants. Interestingly, there is a high probability of interactions between these vitamins, as key pathways and even some individual enzymes use several B vitamins as coenzymes (Asensi-Fabado and Munne-Bosch, 2010). The above information on the role of vitamins B1, B2 and B5 in plants may explain why we have noted their increased accumulation under the influence of the tested iodine compounds. An increase in the level of some B vitamins in plants biofortified with iodoquinolines also results in an improvement in their nutritional value, and thus may have a beneficial effect on the human body.

In this study, we also analyzed the effect of biofortification with various forms of iodine on the content of L-ascorbic acid and dehydroascorbic acid in lettuce. Both AA and DHA are well-known vitamin C, with AA being the reduced form of this vitamin, and DHA being the oxidized form. In plants, ascorbate is oxidized to form, among others, monodehydroascorbate (MDHA) radical, and as a result of spontaneous dismutation of MDHA, DHA is formed. MDHA and DHA regenerate ascorbate through recycling reactions (Locato et al., 2013). Halka et al. (2020) found that the use of KI, 5-ISA and 3,5-diISA resulted in a decrease in the concentration of L-ascorbic acid and an increase in the level of dehydroascorbic acid in tomato leaves. In our study supplementation of lettuce with KIO_3_ and iodoquinolines contributes to both the increase in the content of AA and DHA in most research samples. The increase in DHA content in lettuce leaves biofortified with the studied forms of iodine (except KIO_3_ and 8-hydroxy-7-iodo-5-quinolinesulfonic acid) probably indicates a high degree of oxidation of AA to MDHA, and then to DHA under the influence of these compounds. An increase in the vitamin C content in these plants may increase their defense against oxidative stress, due to its strong antioxidant properties and the presence of an effective system for the regeneration of the redox system (Paciolla et al., 2019). The general strengthening of antioxidant systems is involved in their acclimatization to stress, which is important in post-harvest procedures. Strengthening the antioxidant shield in plant tissues can improve the quality of food during storage (Locato et al., 2013). The content of vitamin C in lettuce may depend not only on the biofortification process (as an exogenous stimulus), but also on many related metabolic processes, such as biosynthesis, recycling, degradation and transport. In addition, the accumulation of this vitamin is influenced by endogenous stimuli (e.g. removal of ROS, synthesis of secondary metabolites) and environmental factors, among which light is of primary importance (Bhullar and Gruissem, 2013; Locato et al., 2013).

Polyphenolic compounds are one of the most widespread and studied groups of plant secondary metabolites. Iodine supplementation can modify their content in plants and thus affect their antioxidant activity (Scarano et al., 2020). Research conducted by Sarrou et al. (2019) showed that the use of different concentrations of KI and KIO_3_ (20, 40 and 80 µM) for the biofortification of sea fennel (*Crithmum maritimum* L.) does not cause significant changes in antioxidant activity. In turn, Sularz et al. (2021) observed an increase in both antioxidant activity and total polyphenol content in lettuce biofortified with 3,5-diISA, while its enrichment with KIO_3_ and 5-ISA also did not affect these parameters. The research results obtained by our team are consistent with the results obtained by Sularz et al. (2021) for lettuce supplemented with 3,5-diISA. All tested iodine compounds contributed to an increase in antioxidant activity (except for plants fertilized with 5-chloro-7-iodo-8-quinolinol and 4-hydroxy-8-iodo-3-quinolinecarboxylic acid) and the total content of polyphenolic compounds. It should be assumed that the increase in the values of these parameters is due to the increased tolerance of plants to abiotic stress caused by the exogenous application of the tested iodine compounds (iodine biofortification) (Zhang et al., 2018). Abiotic stress factors can generate the appearance of secondary stresses, such as oxidative stress, and it is believed that polyphenolic compounds protect the plant against this stress. The action of strong abiotic stress activates defense mechanisms in cells against an increase in the concentration of pro-oxidative factors in them (Blasco et al., 2013; Hajam et al., 2023).

Biofortification with iodine can cause changes in nitrogen metabolism, which in the case of lettuce cultivation are a factor determining its production and quality. Nitrate (V) is the most bioavailable and best absorbed form of nitrogen by plants (Bian et al., 2020). NO_3_^-^ ions are initially reduced to NO_2_^-^ ions by nitrate reductase (NR) and then further reduced to NH_4_^+^ (ammonium ion) by nitrite reductase (NiR) (Miller and Cramer, 2005; de Bang et al., 2021). Weng et al. (2008) and Smoleń et al. (2017) observed a decrease in NO_3_^-^ accumulation, respectively, in water spinach supplemented with iodine in the form of CH_2_ICOO^-^ (iodoacetate) and spinach enriched with KIO_3_ (by soil fertigation) with pre-sowing application of humic and fulvic acids. On the other hand, Smoleń et al. (2022) found that the use of various forms of iodine for biofortification (KIO_3_, KIO_3_ + SA, 5-ISA, 3,5-diISA) does not contribute to significant changes in NO_3_^-^ concentration in lettuce (cultivated on peat substrate and in mineral soil). In the experiment of Blasco et al. (2010) iodine application both in the form of I^-^ and IO_3_^-^ had no significant effect on the concentration of NH_4_^+^ in lettuce. Taking into account the impact of biofortification on the content of NO_2_^-^ ions, it has been shown that the enrichment of lettuce with KIO_3_ contributes to an increase in their concentration (in the case of soil cultivation) (Smoleń et al., 2015a) or does not cause significant changes in their level (in hydroponic cultivation) (Sularz et al., 2020). In our work, the iodine compounds used contributed to the reduction of the content of NO_3_^-^ and NO_2_^-^ and the increase in the concentration of NH_4_^+^ ions in lettuce. The decrease in the concentration of NO_3_^-^ ions could be caused by the fact that the studied forms of iodine affect the process of their absorption by plants or their specific transporters are changed, or there is some kind of antagonism between these forms of iodine and NO_3_^-^ (Blasco et al., 2011a). However, further research is needed to verify these assumptions. In turn, the decrease in the level of NO_2_^-^ and the increase in the content of NH_4_^+^ ions probably results from the increased activity of the NR enzyme (in the first case) and the NiR enzyme (in the second case) under the influence of the tested iodine compounds. It is worth mentioning that NH_4_^+^ can be formed not only as a result of the NO_3_^-^ reduction process, but also when there are high rates of photorespiration due to glycine oxidation (Blasco et al., 2010; Kumari et al., 2022). From the point of view of health safety of potential consumers, the reduction of NO_3_^-^ and NO_2_^-^ content (obtained in these studies) is extremely important, because in the digestive system nitrates are converted into nitrites, and these in turn into carcinogenic N-nitrosamines (Anjana and Iqbal, 2007). In addition, excessive amounts of NO_3_^-^ can impair iodine metabolism in the body by inhibiting iodine uptake by the thyroid, leading to the development of goiter in humans (Garcia Torres et al., 2020).

Previous studies have shown that biofortification with iodine affects the content of chlorides in plants in various ways. Sularz et al. (2020) observed an increase in Cl^-^ concentration in lettuce leaves supplemented with iodosalicylic acids. Smoleń et al. (2015b) found a decrease in the level of these ions in tomato fruits after the application of KI, KIO_3_, KI + SA and KIO_3_ + SA. Our results confirm the results obtained by Smoleń et al. (2015b). The decrease in the concentration of Cl^-^ in lettuce enriched with KIO_3_ and iodoquinolines is probably due to the antagonistic interactions between iodine and chlorine, because chloride channels are easily permeable to iodides and can be transported through these channels (Dávila-Rangel et al., 2019).

In connection with the above information, it should be assumed that the effect of iodine on nitrogen metabolism and chloride content depends, among others, on the form of iodine, plant species and varieties, and growing conditions.

From the point of view of food security, the extent to which biofortified plants meet the human need for iodine, as well as the assessment of the health risk resulting from their consumption, is very important. The RDA-I for adolescents and adults is 150 µg/day (WHO, 2022). The hazard quotient is used to assess the health risk arising from the potential exposure of consumers to chemicals contained in food. Values HQ < 1 indicate a low likelihood of adverse health effects, while values HQ > 1 indicate likely adverse health effects (Kortei et al., 2020). Smoleń et al. (2017) calculated that the consumption of a 50 g portion of fresh spinach leaves biofortified with KIO_3_ with pre-sowing application of humic and fulvic acids (at a concentration of 0.2 cm^3^·dm^-3^ of soil) would provide 406.2 µg I/day (270.8% RDA-I). The same research team estimated that the HQ-I value for a 200 g serving of this vegetable is higher than 1, presumably being a dose dangerous to health. Golob et al. (2020) reported 1.61% RDA-I in 100 g fresh kohlrabi tubers treated with I(V) and an HQ-I value lower than 1 (0.0022). The research results presented by us indicate that the consumption of 50 and 100 g servings of fresh lettuce leaves supplemented with 8-hydroxy-7-iodo-5-quinolinesulfonic acid would provide 293.2 µg I/day (195.46% RDA-I) and 586.39 µg I/day, respectively (390.93% RDA-I). The values obtained are well above the WHO recommended dose, but they are lower than the upper tolerable intake level (UL) for iodine of 1100 µg/day (according to the US Institute of Medicine). It should also be emphasized that the specified UL for iodine applies only to mineral forms of this element (Institute of Medicine, 2001). The maximum allowable intake of organically bound iodine has not yet been established. In our study the HQ-I values for all research objects did not exceed 1, therefore the consumption of this lettuce can be considered potentially safe for the health of consumers.

The use of iodoquinolines to supplement plants with iodine is an economically viable strategy. The wholesale price for 1 kg of pure (99%) iodine compounds is approximately 276 EUR for KIO_3_ and approximately 246 EUR for iodoquinolines. The cost of the total iodine content per 1 kg of fresh marketable weight (FMW) lettuce after the application of KIO_3_, 8-hydroxy-7-iodo-5-quinolinesulfonic acid, 5-chloro-7-iodo-8-quinolinol, 5,7-diiodo-8-quinolinol and 4-hydroxy-8-iodo-3-quinolinecarboxylic acid were 0.044, 0.399, 0.024, 0.045 and 0.035 eurocents per 1 kg of FMW lettuce grown hydroponically, respectively. The cost of biofortification was proportional to the iodine content in the plants.

In this paper, we show for the first time that the exogenous use of iodoquinolines to enrich lettuce with iodine may be a new, improved and sustainable strategy for increasing its nutritional and health-promoting value. Compared to mineral iodine, biofortification of plants with these compounds allows for a higher concentration of iodine and increased levels of most of the assessed nutrients and bioactive compounds. An increase in the content of the above-mentioned ingredients in edible parts of crops may contribute to the improvement of both human health and food security. Reducing iodine deficiency through the use of organoiodine compounds can help achieve the sustainability goal of eliminating hidden hunger, improving nutritional status and promoting sustainable agriculture. Nevertheless, knowing the amount of iodine in lettuce biofortified with iodoquinolines is insufficient to predict how it might meet human nutritional requirements. Therefore, *in vivo* studies evaluating the bioavailability of iodine from these plants are needed to determine its effect on the nutritional status of the body. The biostimulating effect of iodoquinolines on the growth of lettuce makes the cultivation of plants biofortified in this way more attractive for farmers due to higher yields. The additional increase in the content of secondary metabolites (polyphenolic compounds) after the use of iodoquinolines, due to the increase in stress tolerance of these plants, is an important strategy that can meet the nutritional needs of the population. Moreover, stress-resistant crops are needed to ensure crop stability under stress conditions and to minimize the environmental impact of crop production. Our research has also shown that lettuce supplemented with organoiodine compounds is characterized by a much lower concentration of nitrates (V) and nitrates (III), which is extremely important due to their participation in the formation of carcinogenic N-nitrosamines. Lettuce was grown hydroponically, i.e. in a way that protects and improves the condition of the natural environment thanks to, among others, high efficiency of water use and reduction of fossil fuels (crude oil). Environmental protection is an essential prerequisite for sustainable agriculture. The benefits resulting from the use of iodoquinolines for biofortification of these plants, such as: 1) improvement of nutritional and health-promoting quality, 2) biostimulating effect on growth and 3) increasing the resistance of the crop to stress make it possible to consider their implementation in agricultural practice. In addition, combating the problem of iodine deficiency in the human diet and strengthening sustainable plant production through biofortification and biostimulation of lettuce with iodoquinolines fits very well with the challenges posed by climate protection agriculture.

## 5 Conclusion

**Figure 1:**
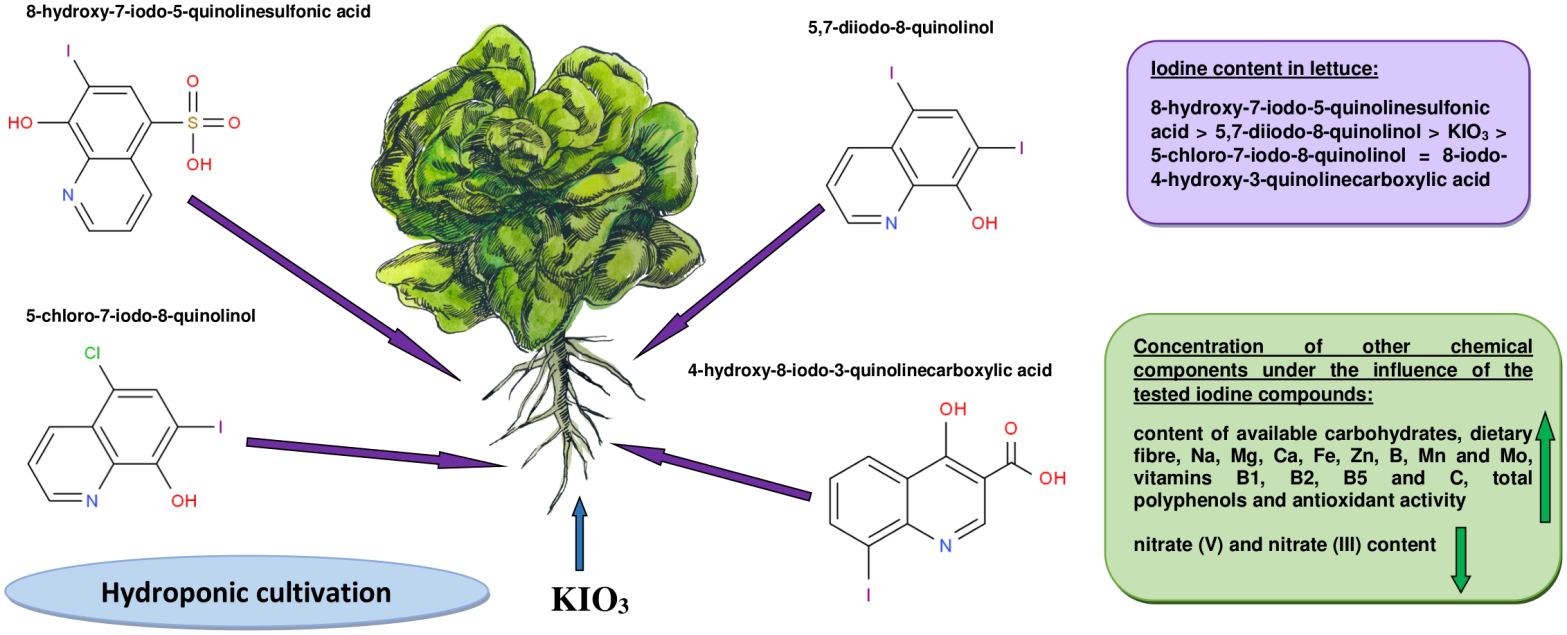
Lettuce biofortification with iodoquinolines versus KIO_3_.

The research results contained in this publication clearly indicate that the use of iodoquinolines for lettuce biofortification may be a potential sustainable strategy to combat malnutrition caused by iodine deficiency, as well as lead to improved human health. These organoiodine compounds can be a source of iodine for plants and have a more favorable effect on their chemical composition than inorganic iodine. Compared to KIO_3_, a higher content of iodine, and thus the percentage of RDA, was found in plants supplemented with 8-hydroxy-7-iodo-5-quinolinesulfonic acid and 5,7-diiodo-8-quinolinol (**Figure 1**). All tested iodine compounds had a biostimulating effect on lettuce yield, especially 5-chloro-7-iodo-8-quinolinol, after application of which the highest weight of the whole plant and its head was noted. Lettuce enriched with most of these compounds was characterized by a higher content of assimilable carbohydrates, dietary fiber, vitamins B1, B2 and B5, vitamin C and minerals such as Na, Mg, Ca, Fe, Zn, Mn, Mo and B (compared to control lettuce and enriched with mineral iodine). In addition, after their use, a significant increase in antioxidant activity and total polyphenol concentration was also observed. Biofortification with organic forms of iodine contributed to the reduction of the concentration of nitrates (V) and nitrates (III), and the obtained HQ-I values for all research objects were lower than 1. Among the tested iodine compounds, the use of 5,7-diiodo-8-quinolinol and 8-hydroxy-7-iodo-5-quinolinesulfonic acid for supplementation had the most beneficial effect on most of the analyzed parameters of the chemical composition of lettuce.

## Data availability statement

The original contributions presented in the study are included in the article/**Supplementary Material**. Further inquiries can be directed to the corresponding authors.

## Author contributions

AD: Data curation, Formal analysis, Funding acquisition, Investigation, Methodology, Visualization, Writing – original draft. SS: Investigation, Methodology, Validation, Writing – review & editing. AW-Ś: Investigation, Methodology, Writing – review & editing. IK: Investigation, Methodology, Writing – review & editing. OS: Investigation, Methodology, Writing – review & editing. JK: Investigation, Methodology, Writing – review & editing. JP: Investigation, Methodology, Writing – review & editing. AK: Conceptualization, Formal analysis, Funding acquisition, Methodology, Supervision, Validation, Writing – review & editing.
