# Supplementary material for: Evaluation of the chemical composition and nutritional value of lettuce (Lactuca sativa L.) biofortified in hydroponics with iodine in the form of iodoquinolines
Source: Front Plant Sci. 2023 Nov 23;14:1288773. doi: 10.3389/fpls.2023.1288773 (PMC10701912; doi:10.3389/fpls.2023.1288773)
Supplement: Supplementary file 1 [file DataSheet_1.docx]

Supplementary Material

Evaluation of the chemical composition and nutritional value of lettuce (*Lactuca sativa* L.) biofortified in hydroponics with iodine in the form of iodoquinolines

Agnieszka Dyląg^1*^, Sylwester Smoleń^2^, Anna Wisła-Świder^3^, Iwona Kowalska^2^, Olga Sularz^1^, Joanna Krzemińska^1^, Joanna Pitala^4^ and Aneta Koronowicz^1*^

^1^Department of Human Nutrition and Dietetics, Faculty of Food Technology, University of Agriculture in Krakow, Krakow, Poland

^2^Department of Plant Biology and Biotechnology, Faculty of Biotechnology and Horticulture, University of Agriculture in Krakow, Krakow, Poland

^3^Department of Chemistry, Faculty of Food Technology, University of Agriculture in Krakow, Krakow, Poland

^4^Laboratory of Mass Spectrometry, Faculty of Biotechnology and Horticulture, University of Agriculture in Krakow, Krakow, Poland

*** Correspondence:**Agnieszka Dyląg, agnieszka.dylag@poczta.fm; Aneta Koronowicz, aneta.koronowicz@urk.edu.pl

**Supplementary Figure 1.** Reaction scheme for 5,7-diiodo-8-quinolinol synthesis


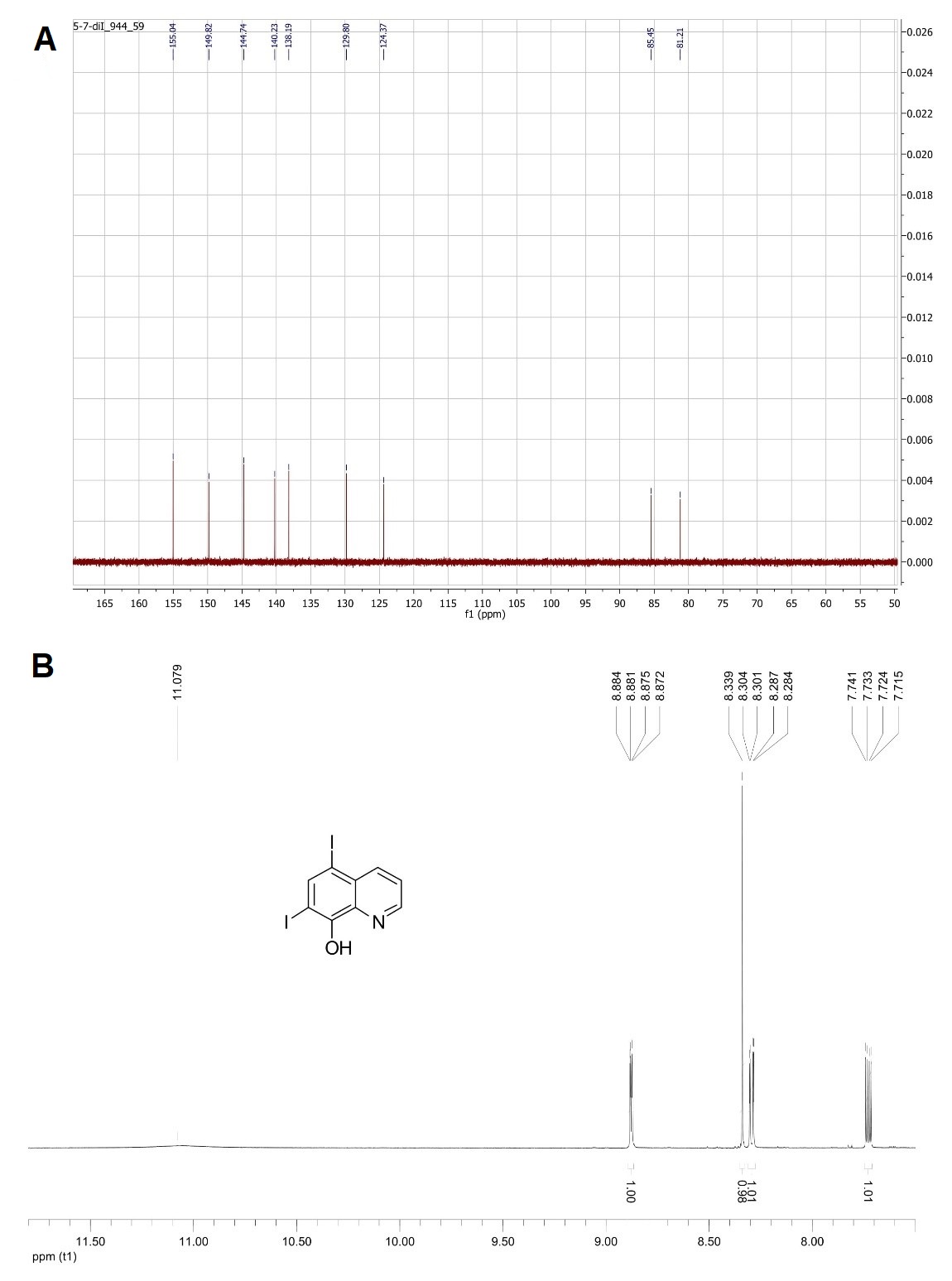


**Supplementary Figure 2.** ^1^H NMR (A) and ^13^C NMR (B) spectra of 5,7-diiodo-8-quinolinol.

Yield: 78 %; mp = 210-214°C.^1^H NMR (500 MHz, DMSO-d_6_) δ 11.08 (br s, 1H, OH), 8.88 (dd, 1H, *J* = 4.5, 1.5 Hz, H-2), 8.34 (s, 1H, H-6), 8.29 (dd, 1H, *J* = 8.5, 1.5 Hz, H-4), 7.73 (dd, 1H, *J* = 8.5, 4.5 Hz, H-3); ^13^C NMR (125 MHz, DMSO-d_6_) δ 155.04 (C-8), 149.82, 144.74, 140.23, 138.19, 129.80, 124.37, 85.45 (C-5), 81.21 (C-7). Standard abbreviations indicating multiplicity were as follows: s = singlet, d = doublet, dd = doublet of doublets, t = triplet, and br = broad.
